# Exopolysaccharides Play a Role in the Swarming of the Benthic Bacterium *Pseudoalteromonas* sp. SM9913

**DOI:** 10.3389/fmicb.2016.00473

**Published:** 2016-04-05

**Authors:** Ang Liu, Zi-Hao Mi, Xiao-Yu Zheng, Yang Yu, Hai-Nan Su, Xiu-Lan Chen, Bin-Bin Xie, Bai-Cheng Zhou, Yu-Zhong Zhang, Qi-Long Qin

**Affiliations:** ^1^Marine and Agricultural Biotechnology Laboratory, State Key Laboratory of Microbial Technology, Shandong UniversityJinan, China; ^2^Marine Biotechnology Research CenterJinan, China

**Keywords:** exopolysaccharide, swarming, marine sediment, benthic bacteria, flagella

## Abstract

Most marine bacteria secrete exopolysaccharide (EPS), which is important for bacterial survival in the marine environment. However, it is still unclear whether the self-secreted EPS is involved in marine bacterial motility. Here we studied the role of EPS in the lateral flagella-driven swarming motility of benthic bacterium *Pseudoalteromonas* sp. SM9913 (SM9913) by a comparison of wild SM9913 and Δ*epsT*, an EPS synthesis defective mutant. Reduction of EPS production in Δ*epsT* did not affect the growth rate or the swimming motility, but significantly decreased the swarming motility on a swarming plate, suggesting that the EPS may play a role in SM9913 swarming. However, the expression and assembly of lateral flagella in Δ*epsT* were not affected. Instead, Δ*epsT* had a different swarming behavior from wild SM9913. The swarming of Δ*epsT* did not have an obvious rapid swarming period, and its rate became much lower than that of wild SM9913 after 35 h incubation. An addition of surfactin or SM9913 EPS on the surface of the swarming plate could rescue the swarming level. These results indicate that the self-secreted EPS is required for the swarming of SM9913. This study widens our understanding of the function of the EPS of benthic bacteria.

## Introduction

Bacterial exopolysaccharide (EPS) is mostly generated intracellularly and then exported to the extracellular environment (Ates, 2015). Most marine bacteria are surrounded by self-secreted EPS, which is believed to be of great importance to the survival of marine bacteria in various oceanic environments (Nichols et al., 2005; Poli et al., 2010). Marine bacterial EPS can act as a cryoprotectant to help bacteria adapt to the extreme low temperature in sea ice (Nichols et al., 2005; Carrion et al., 2015), aid bacteria in absorbing nutrient elements (Decho and Lopez, 1993; Guezennec, 2002; Nichols et al., 2005), and assist bacteria in attaching to organic particles and other surfaces (Fletcher and Floodgate, 1973; Paerl, 1975; Holmstrom and Kjelleberg, 1999). However, it is still unknown whether self-secreted EPS is involved in the motility of marine bacteria.

Bacteria have several motile patterns. Swarming is a kind of rapid surface translocation powered by flagella, and is a typical pattern of surface-associated motile lifestyle (Henrichsen, 1972; Kearns, 2010). Some bacteria have been reported to have swarming motility, such as strains in *Proteus* (Mobley and Belas, 1995), *Vibrio* (McCarter and Silverman, 1990), *Bacillus* (Kearns and Losick, 2003), *Pseudomonas* (Kohler et al., 2000), *Escherichia* (Harshey, 1994), *Salmonella* (Harshey, 1994), *Serratia* (Eberl et al., 1999), *Aeromonas* (Kirov et al., 2002), *Azospirillum* (Hall and Krieg, 1983), *Clostridium* (Hernandez and Rodriguez, 1993), *Rhodospirillum* (Ragatz et al., 1995), and *Yersinia* (Young et al., 1999). The roles of EPS in the swarming of several non-marine bacteria have been studied, but the results are different, even controversial, in different strains. In *Sinorhizobium meliloti* RMB7201, fast-swarming mutants were found to synthesize less EPS and all EPS-I overproducing mutants had swarming defects in different degrees (Wei and Bauer, 1999). A mutant of *Pseudomonas aeruginosa* with increased swarming motility was also found to have defect in EPS production (Merritt et al., 2007). In contrast, the EPS defective mutants of *Bacillus subtilis* could not swarm (Nagorska et al., 2010). The EPS II defective strain of *S. meliloti* Rm8530 was also found to be unable to swarm (Gao et al., 2012). Therefore, it is necessary to further examine the effect of self-secreted EPS on bacterial swarming motility. The role of self-secreted EPS in the swarming motility of marine bacteria has not been reported. Determining the relationship of the EPS and the swarming motility of marine bacteria would broaden our understanding of marine bacteria.

*Pseudoalteromonas* sp. SM9913 (hereafter called SM9913) is a marine bacterium isolated from the deep-sea sediment of Okinawa Trough and noted for its good extracellular protease-producing ability (Chen et al., 2003; Zhao et al., 2008; Yan et al., 2009). Due to its good extracellular protease-producing ability, SM9913 may play a role in organic nitrogen degradation in deep-sea sediment (Zhao et al., 2008). SM9913 also secretes a large amount of EPS (Qin et al., 2007). The EPS of SM9913 has been shown to have several ecological roles, such as stabilizing the protease secreted by the same strain and binding metal ions, which may be important for the survival of SM9913 in the extreme sedimentary environment (Qin et al., 2007). It was also found that the EPS of SM9913 played a role in the attachment of SM9913 cells to clay particles (Qin et al., 2007; Mi et al., 2015). Recently, SM9913 was found to have lateral flagella propelled swarming motility, which is helpful for the strain to survive in the deep-sea sediment (Mi et al., 2015). However, it is still unknown whether the EPS of SM9913 has an effect on its swarming motility.

During bacterial EPS biosynthesis, a majority of EPS share an initiation step requiring a UDP sugar and the undecaprenyl-phosphate (Und-P) lipid carrier (Whitfield, 2006). In this step, UDP-glucose lipid carrier transferase transfers a UDP sugar to a repeating unit attached to glycosyl carrier lipid and catalyzes the formation of a phosphoanhydride bond between them. Then the repeating units are polymerized, and the synthesized polysaccharide is secreted across membranes and cell wall (Ates, 2015). Therefore, UDP-glucose lipid carrier transferase is essential for bacterial EPS biosynthesis. In the genome of SM9913, a EPS gene cluster is predicted to contain 21 genes (from gene PSM_A1265 to PSM_A1287) and the gene *epsT* (PSM_A1282) encodes the initial UDP-glucose lipid carrier transferase (Qin et al., 2011). We have deleted the gene *epsT* from SM9913 and an EPS synthesis defective mutant Δ*epsT* was obtained. The EPS production of Δ*epsT* was only 27% of that of wild SM9913 (Yu et al., 2014). The reduction of EPS production severely decreased the particle-attached ability of Δ*epsT*, suggesting that the EPS secreted by SM9913 is important in the attachment of SM9913 to sedimentary particles (Mi et al., 2015). In this study, wild SM9913 and Δ*epsT* were compared to investigate whether the EPS of SM9913 plays a role in the swarming motility of the strain. Our results indicate that the reduction of EPS production in Δ*epsT* significantly decreased the swarming motility on a swarming plate. Further analysis showed that the self-secreted EPS may facilitate SM9913 swarming by increasing the wetness of colony brim and reduce the surface tension.

## Materials and Methods

### Strains and Culture

SM9913 was previously isolated from the deep-sea sediment of Okinawa Through (Chen et al., 2003). Δ*epsT*, an EPS biosynthesis defective mutant of SM9913, was previously constructed by deleting the *epsT* gene encoding the initial UDP-glucose lipid carrier transferase in the EPS gene cluster (Yu et al., 2014). The complement strain Δ*epsT*/pEV*eps*T was previously constructed by introducing a multicopy plasmid carrying the *epsT* gene into Δ*epsT*, and the control strain Δ*epsT*/pEV was previously constructed by introducing the empty multicopy plasmid into Δ*epsT* (Mi et al., 2015). These bacterial strains were grown in marine LB (Luria-Bertani) medium at 15°C as previously reported (Qin et al., 2011; Yu et al., 2014). The artificial sea water was prepared as described previously (Mi et al., 2015). To construct the growth curves of SM9913 and Δ*epsT*, strains were cultured in marine LB at 15°C, and the optical density at 600 nm was measured with the time interval of 1 h using the Bioscreen C MBR microbial growth monitoring instrument (Oy Growth Curves Ab, Finland).

### Swimming and Swarming Assays

The swimming and swarming motilities of SM9913 and Δ*epsT* were assayed as previously described (Mi et al., 2015). For swarming assay, 20 ml of marine LB medium solidified by 0.5% agar (Bacto^TM^ agar, USA) were poured into each petri dish (Biologix, US) and the plates were dried under vertical flow for 50 min. For the complemented strains, chloramphenicol was added to a final concentration of 20 μg/ml. To evaluate swarming motility, overnight cultures were adjusted to the same optical density of OD_600_ 1.5 and 5 μl of the cultures were spotted on each plate. The plates were incubated at 15°C for 5 days and motility was measured by examining the migration of bacteria through the agar from the center toward the periphery of the plate. For the swimming assay, 20 ml of marine LB medium solidified by 0.3% agar (Bacto^TM^ agar, USA) were poured into each petri dish (Biologix, US) and the plates were dried under vertical flow for 50 min. Overnight cultures were adjusted to the same optical density of OD_600_ 1.5 and 5 μl of the cultures were spotted on each plate. The plates were incubated at 15°C for 3 days.

### Comparison of the Expression Levels of Genes in the EPS Gene Cluster between the Swarming and Swimming Cells of SM9913

RNA sequencing of the swarming and swimming cells of SM9913 was previously performed (Mi et al., 2015). The swimming cells for RNA sequencing were prepared by growing SM9913 in liquid marine LB to the optical density at 600 nm (OD_600_) of approximately 1.0. The swarming cells for RNA sequencing were taken from the edge of swarming colonies with a sterile cotton swab (Mi et al., 2015). In this study, based on the RNA sequencing data, the expression levels of genes (from PSM_A1265 to PSM_A1287, among which PSM_A1277 and PSM_A1278 were pseudogenes) in the EPS gene cluster were calculated using the *RPKM* method (Mortazavi et al., 2008) and compared between the swarming and swimming cells. The comparison was indicated as relative expression level = log_2_(swarming/swimming). A gene was regarded as differentially expressed when its log_2_(swarming/swimming) ≥ 1 and when its *P* ≤ 0.001.

### Real-Time Quantitative PCR

The expression of four genes involved in the biosynthesis of lateral flagella in the lateral flagella gene cluster in wild SM9913 and Δ*epsT* was examined by real-time quantitative PCR. The total RNA was extracted from SM9913 and Δ*epsT* as described previously (Mi et al., 2015). The cells for RNA extraction were harvested by sterile cotton swabs from the edge of swarming colonies after 72 h incubation. Reverse transcription was performed using the PrimeScript^TM^ RT reagent kit with gDNA Eraser (perfect Real Time; TaKaRa, Japan). The obtained cDNA and the SYBR^®^*Premix Ex Taq*^TM^ (Tli RNaseH plus; TaKaRa, Japan) were used in preparing the quantitative PCR reaction system and real-time quantitative PCR was performed on LightCycler 480^®^(Roche, Switzerland). Fold change of the expression level of target genes was calculated by the software in LightCycler 480^®^with *rpoD* as the reference gene. The extraction of total RNA and the subsequent quantitative PCR were repeated three times with three replicates. Primers used for PCR are listed in **Table 1**.

**Table 1:** Primers used in this study.

Primer	Sequence (5′–3′)	Target gene
*rpoD*F	CGCATATTATTGACTGGTTAGGTG	*rpoD*
*rpoD*R	CAAGGGTTGAGGGTTCATAGC	
RT0917F	CTCGTTCTGTGTCGGTGGTG	PSM_A0917
RT0917R	TTTATCGGTTTCTTTGCCTACTGG	
RT0915F	GGCATCGCACCCAAACAG	PSM_A0915
RT0915R	ATCAATATCAATACCGCATTCACG	
RT0906F	ATTACGAGCCAGAGTCAGTTCAG	PSM_A0906
RT0906R	CCACACCACCCACGCTAATG	
RT0897F	GGCTGGTCTACAAATCGCTTCAC	PSM_A0897
RT0897R	TCATTTCATCAA‘ACGCACCTTCAG	

### Atomic Force Microscopy

Lateral flagella of SM9913 and Δ*epsT* were observed by using an atomic force microscope (AFM) as previously reported (Mi et al., 2015). Briefly, cells were taken from the edge of a swarming colony and suspended in distilled water. The obtained cell suspension was imaged using an AFM (Bruker AXS, Germany).

### Quantification of Swarming Behavior

To measure the diameter of each colony, a line was drawn on the bottom side of the petri dish across the center of each colony and the diameter was measured along the drawn line. Data of diameters were recorded and plotted against time. The obtained plots were fitted using growth equation for SM9913 and Δ*epsT*. Rate of colony expansion was calculated by differentiating diameter with respect to time using the fitted curves. Line fitting and curve differentiating were performed using the software OriginPro 8. The experiment was repeated three times with at least three replicates. *P*-value was calculated from *t*-test.

### Preparation of EPS from SM9913

The EPS of SM9913 was prepared with the method of Liu et al. (2013). Briefly, marine LB medium with 2% inoculum was incubated at 15°C, 200 rpm for 5 days. After fermentation, EPS in the supernatant of the culture was precipitated with chilled absolute ethanol. The precipitate was dissolved in distilled water. Proteins in the EPS solution were removed by the sevag method (Staub, 1965), and small molecular carbohydrates were removed by dialyzing the EPS solution against distilled water. The polysaccharide content of the EPS solution was determined using the phenol-sulfuric acid method.

### Rescue of the Swarming Defect of Δ*epsT* with Surfactin and the EPS from Wild SM9913

Surfactin from *B. subtilis* (Sigma, USA) was dissolved in 20 mM NaOH. Five microliters of surfactin (2 mg/ml) or the EPS extracted from wild SM9913 (2 mg/ml) were spotted onto swarming plates and air dried. Then, 5 μl of the overnight culture of Δ*epsT* or wild SM9913 was spotted on the location of surfactin or EPS. The inoculated plates were incubated at 15°C for 5 days. The measurement of the diameter against time was performed as described above. The experiment was repeated three times with at least three replicates. *P*-value was calculated from *t*-test.

## Results

### Reduction of EPS Production Caused a Decrease in the Swarming Motility of SM9913

**FIGURE 1:**
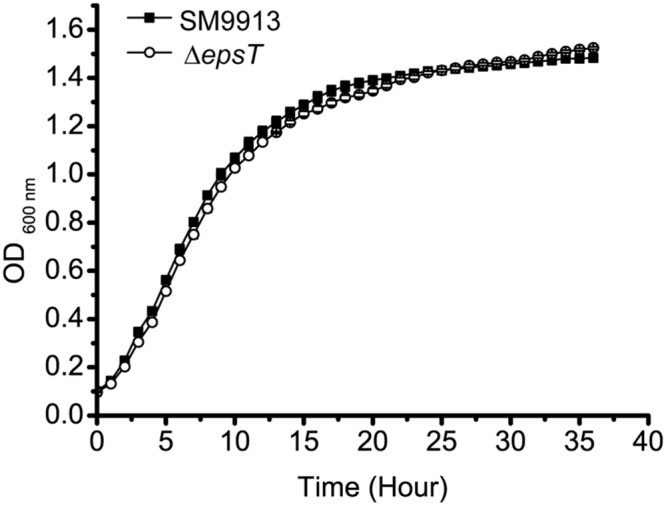
**The growth of wild SM9913 and Δ*epsT* in marine LB medium.** Each plot represents the mean of five parallel samples (mean ± SD).

Δ*epsT* is an EPS biosynthesis defective mutant of SM9913 with the *epsT* gene being knocked out and 73% reduction of EPS production (Yu et al., 2014). To investigate whether the reduction of EPS production affects the growth of SM9913, the growth of wild SM9913 and Δ*epsT* was compared. As shown in **Figure 1**, Δ*epsT* displayed a similar growth rate as wild SM9913, indicating that the reduction of EPS production does not affect the growth of SM9913.

**FIGURE 2:**
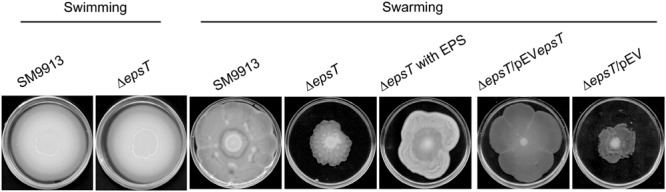
**The swimming and swarming motilities of wild SM9913, Δ*epsT*, Δ*epsT*/pEV*epsT*, and Δ*epsT*/pEV.** For swimming assay, cultures of strains were inoculated onto the centers of plates containing 0.3% agar and the inoculated plates were incubated at 15°C for 3 days. For swarming assay, cultures of strains were inoculated onto the centers of plates containing or 0.5% agar and the inoculated plates were incubated at 15°C for 5 days. For the assay of Δ*epsT* with exopolysaccharide (EPS), 5 μl of EPS (2 mg/ml) were dropped onto the center of the plate before inoculation. Pictures are a representative of three independent experiments.

SM9913 has both swimming and swarming motilities (Mi et al., 2015). To study the role of the EPS in the motilities of SM9913, the swimming and swarming motilities of wild SM9913 and Δ*epsT* were compared. On the swimming plates, wild SM9913 and Δ*epsT* displayed similar swimming motility (**Figure 2**), indicating that the reduction of EPS had no effect on the swimming motility of SM9913. In contrast, the swarming colony of Δ*epsT* was much smaller than that of wild SM9913 after 5-day incubation (**Figure 2**), indicating a lower swarming motility of Δ*epsT* than wild SM9913. In addition, the swarming motility of the complement strain Δ*epsT*/pEV*epsT* could be rescued to the level of wild SM9913 by re-introducing the gene *epsT* into Δ*epsT*, but the swarming of the control strain Δ*epsT*/pEV was not rescued (**Figure 2**). Moreover, when the EPS extracted from SM9913 was spotted in advance on the location where Δ*epsT* was inoculated on the swarming plate, the swarming defect of Δ*epsT* could be rescued to a large extent (**Figure 2**). EPS is a polymer and is difficult to diffuse in the agar on the swarming plate, which may be the main reason why the swarming defect of Δ*epsT* was not completely rescued by the EPS from SM9913. Altogether, these results showed that the reduction of EPS production in Δ*epsT* affected its swarming, which suggests that the self-secreted EPS of SM9913 may play a role in its swarming.

### EPS Production in SM9913 Was Not Coupled with Swarming

**FIGURE 3:**
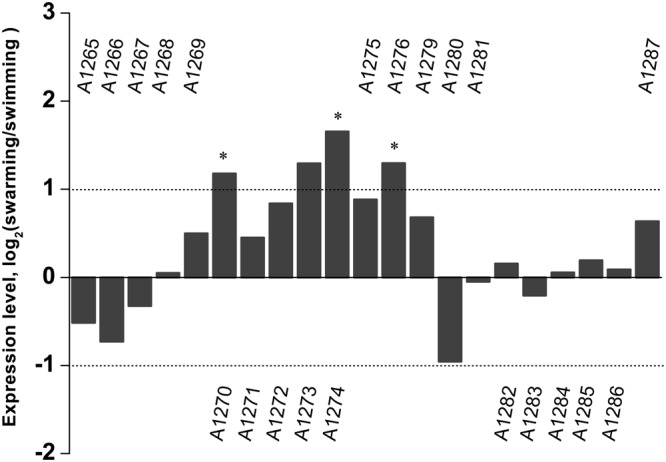
**The relative expression levels of genes belonging to the EPS gene cluster between swarming and swimming cells of SM9913 based on transcriptome data.** Differentially expressed genes were indicated by the symbol ^∗^. A gene was regarded as a differentially expressed gene when its | log_2_(swarming/swimming)|≥ 1 (demarcated by dot lines) and its *P* ≤ 0.001. Gene PSM_A1273 was not regarded as a differentially expressed gene because its *P*-value was greater than 0.001.

Transcriptome sequencing of the swarming and swimming cells of SM9913 were previously performed (Mi et al., 2015). To analyze whether the production of EPS in SM9913 is coupled with swarming, the expression levels of genes in the EPS gene cluster were compared between the swarming and swimming cells according to the transcriptome data. The results showed that, while 3 of the 21 genes in the EPS gene cluster were differentially expressed slightly, the other 18 genes (including the deleted gene *epsT*) did not show differential expression between the swarming and swimming cells (**Figure 3**). This suggests that the expression of genes in the EPS gene cluster is not coupled with the regulation of swarming, and that the swarming of SM9913 does not induce EPS production.

### Deletion of Gene *epsT* Did Not Affect the Biosynthesis of Lateral Flagella in SM9913

**FIGURE 4:**
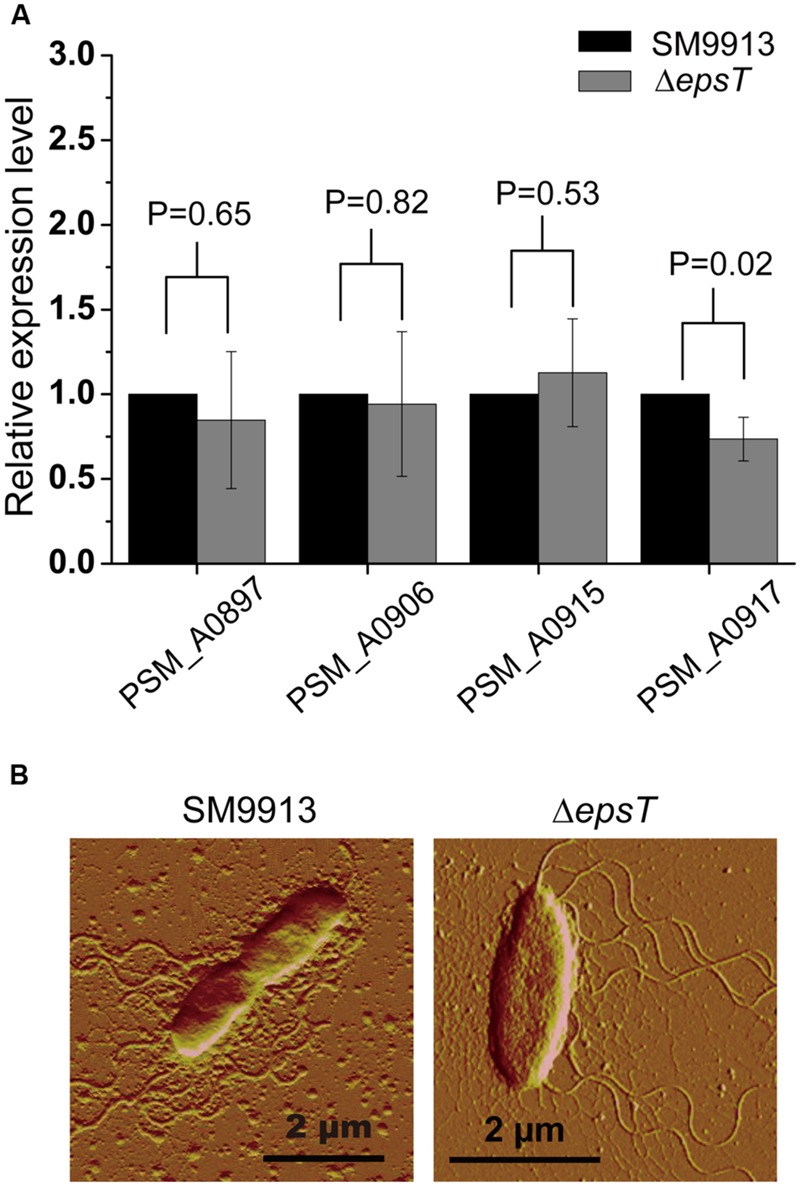
**Comparisons of lateral flagella biosynthesis between Δ*epsT* and wild SM9913 swarming cells. (A)** The relative expression levels of four lateral flagella genes in Δ*epsT* and wild SM9913 swarming cells analyzed by real-time quantitative PCR. The data were from three independent experiments with three replicates (mean ± SD). *P*-value of each gene was calculated from *t*-test and was indicated. **(B)** The lateral flagella of wild SM9913 and Δ*epsT* observed by atomic force microscopy. Cells were taken from the edge of a swarming colony and imaged by atomic force microscopy.

SM9913 uses lateral flagella to power its swarming motility (Mi et al., 2015). To investigate whether the deletion of gene *epsT* affected the biosynthesis of lateral flagella in SM9913, the expression of four genes involved in the biosynthesis of lateral flagella in the lateral flagella gene cluster in wild SM9913 and Δ*epsT* was examined by real-time quantitative PCR. The result showed that the expression of these four genes displayed no significant difference between wild SM9913 and Δ*epsT* (**Figure 4A**), which indicated that the deletion of gene *epsT* did not affect the biosynthesis of lateral flagella in SM9913. An AFM observation of their lateral flagella also supported this. The lateral flagella of Δ*epsT* were clearly viewed under AFM (**Figure 4B**). These results indicate that the expression and assembly of lateral flagella in Δ*epsT* are not affected and that the decrease of the swarming motility of Δ*epsT* is not due to the lack of lateral flagella. Alternatively, EPS reduction in Δ*epsT* may affect the function of lateral flagella.

### EPS Reduction in Δ*epsT* Caused Changes in the Swarming Behavior

**FIGURE 5:**
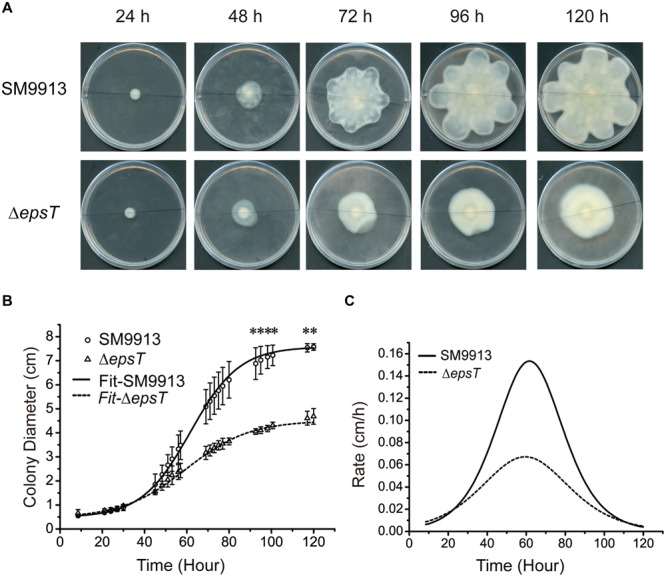
**Analyses of the swarming modes of wild SM9913 and Δ*epsT*. (A)** Swarming colonies of SM9913 and Δ*epsT* at different incubation time. Pictures are a representative of three independent experiments. **(B)** Colony expansion curves of wild SM9913 and Δ*epsT* fitted with growth equation based on measured colony diameters (scatters). Data were from three independent experiments with at least three replicates (mean ± SD). *P*-value was calculated from *t*-test and the symbol ^∗^ indicates that the difference in colony diameter between wild SM9913 and Δ*epsT* was significant (*P* < 0.001). **(C)** Colony expansion rates of wild SM9913 and Δ*epsT*. Curves were obtained by differentiating diameter with respect to time using fitted curves.

To analyze the possible change in the swarming behavior of Δ*epsT* caused by EPS reduction, we quantified the swarming of wild SM9913 and Δ*epsT* by recording the diameters of swarming colonies with incubation time and calculated their expansion rates. The result showed that the diameter of the swarming colony of Δ*epsT* was smaller than that of SM9913 after 120-h incubation (**Figure 5A**). Statistical analysis indicated that the difference in colony diameter between wild SM9913 and Δ*epsT* was significant (*P* < 0.001). Moreover, it can be seen from **Figures 5A,B** that the colony expansion modes between wild SM9913 and Δ*epsT* were also different. During the first 30 h of incubation, diameters of wild SM9913 and Δ*epsT* colonies increased very slowly (**Figure 5B**), showing an obvious swarming lag. After the lag period, the colony of wild SM9913 expanded quickly. The colony expansion rate increased with time, reached the maximum (0.16 cm/h) at the 65th hour, and then declined (**Figure 5C**). In contrast, the colony of Δ*epsT* did not show an obvious rapid expansion period. Its expansion rate increased almost constantly from the beginning, reached a much lower maximum (approximately 0.06 cm/h) at the 60th hour, and then declined (**Figure 5C**). Thus, Δ*epsT* finally formed a much smaller colony compared to the wild strain (**Figure 5A**). These results indicated that EPS reduction led to a change in the swarming behavior of SM9913, which caused the decrease of its swarming motility on the swarming plate.

### The Rescue of Δ*epsT* Swarming by Extracellularly Added Biosurfactant Surfactin

**FIGURE 6:**
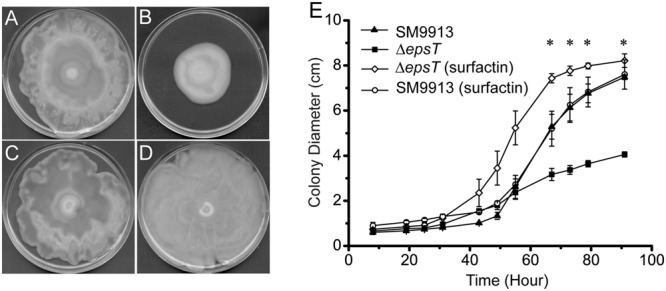
**The rescue of the swarming of Δ*epsT* with surfactin. (A)** Wild SM9913 on a swarming plate without surfactin. **(B)** Δ*epsT* on a swarming plate without surfactin. **(C)** Wild SM9913 on a swarming plate with surfactin. **(D)** Δ*epsT* on a swarming plate with surfactin. Five microliters of surfactin (2 mg/ml) were dropped onto the center of the plate before inoculation. **(E)** Colony diameters of wild SM9913 in **(A)** and **(C)** and Δ*epsT* in **(B)** and **(D)**. Data were from three independent experiments with at least three replicates (mean ± SD). *P*-value was calculated from *t*-test and the symbol ^∗^ indicates that the difference in colony diameter between Δ*epsT* with or without surfactin was significant (*P* < 0.001). Pictures are a representative of three independent experiments.

Surfactin is a biosurfactant from *B. subtillis*. We investigated whether surfactin could rescue the swarming of Δ*epsT*. As shown in **Figure 6**, the colony diameter of Δ*epsT* on the plate with surfactin was significantly larger than that of this mutant without surfactin after 91 h incubation (*P* < 0.001) and similar to that of wild SM9913 (**Figures 6A,B,D,E**), indicating that the addition of surfactin could rescue the swarming of Δ*epsT*. However, surfactin added on the plate had no effect on the swarming of wild SM9913 (**Figures 6A,C**).

## Discussion

The results in this study indicated that the EPS of SM9913 had a positive effect on its swarming motility, which is consistent with the role of EPS on the swarming motilities of *B. subtilis* (Nagorska et al., 2010) and *S. meliloti* Rm8530 (Gao et al., 2012). Bacterial swarming motility is driven by rotating flagella and coupled to the production of a mucoid layer that facilitates the movement (Verstraeten et al., 2008; Kearns, 2010). The mucoid layer serves as wetting agents to extract water from the surroundings and also as surfactants to reduce tension between the substrate and the bacterial cells at the swarming front (Verstraeten et al., 2008; Kearns, 2010). The swarming of SM9913 is driven by its lateral flagella (Mi et al., 2015). In its mutant Δ*epsT*, the expression and assembly of lateral flagella was not altered (**Figure 4**), but the EPS production was severely reduced (Yu et al., 2014), which would affect the mucoid layer. This is likely the main cause leading to the swarming defect of Δ*epsT*. In the meantime, the possibility that the loss of the *espT* gene in Δ*epsT* has other effects on the swarming behavior that is independent of EPS production can not be excluded completely, which needs further study.

Reports on the mechanistic benefit of EPS during bacterial swarming are quite limited. It was suggested that during *Escherichia coli* swarming, lipopolysaccharides (LPS) likely functioned as osmolytes facilitating water extraction from the agar, which then advantaged the swarming motility (Ping et al., 2014). Bacterial capsular exopolysaccharides (CPS) were also suggested to facilitate the translocation over solid surfaces of differentiated cell populations by aiding colony hydration (Gygi et al., 1995; Rahman et al., 1999). Thus, although bacterial LPS and CPS have differences in their characteristics, they seems to play similar roles in bacterial swarming motility, increasing the wetness of colony brim to provide a liquid environment for flagella to function properly (Gygi et al., 1995; Rauprich et al., 1996; Toguchi et al., 2000; Chen et al., 2007). Therefore, the EPS of SM9913 may also play a similar role in its swarming motility, increasing the wetness of colony brim to provide a liquid environment for flagella movement, and thereby facilitating the swarming of SM9913.

Some species, such as *B. subtilis*, secrete both surfactin as a surfactant and EPS as a wetting agent to facilitate its swarming motility (Nagorska et al., 2010). SM9913 secretes a large amount of EPS (Qin et al., 2007), but does not secrete surface-active agent (data not shown). Surfactin is a kind of amphipathic molecules. Surfactin can improve the wettability of the substrate, and subsequently reduce surface tension, which permit bacteria spreading over surfaces (Julkowska et al., 2004; Ke et al., 2015). It is reported that surfactin was able to rescue the swarming of LPS biosynthesis defective mutant in *Salmonella enterica* (Toguchi et al., 2000). Similar to the effect of surfactin on *S. enterica* (Toguchi et al., 2000), our results showed that surfactin could completely compensate the swarming defect of Δ*epsT* by improving the colony expanding rate, but had little effect on the swarming motility of wild SM9913 (**Figure 6**). This implies that the EPS of SM9913 may be also helpful in reducing the surface tension in SM9913 swarming.

In summary, our results in this study show that the self-secreted EPS of benthic strain SM9913 plays a positive role in its swarming motility. The EPS may facilitate SM9913 swarming by increasing the wetness of colony brim and reducing the surface tension. This study sheds more light on the function of the EPS of benthic bacteria.

## Author Contributions

AL, Z-HM, and X-LC designed, conducted and wrote the paper. X-YZ, YY, and H-NS conducted the experiments. B-BX, B-CZ, and Y-ZZ analyzed the data. Q-LQ instructed the study and revised the paper.

## Conflict of Interest Statement

The authors declare that the research was conducted in the absence of any commercial or financial relationships that could be construed as a potential conflict of interest.
